# Loss of SENP3 mediated the formation of nasal polyps in nasal mucosal inflammation by increasing alternative activated macrophage

**DOI:** 10.1002/iid3.781

**Published:** 2023-02-09

**Authors:** Ximing Bao, Bin Liu, Yongquan Jiang, Tingting Feng, Wanxin Cao, Jiali Shi, Yiming Jiang, Xiaorui Chen, Jie Yang, Jiping Li, Zheng Zhou

**Affiliations:** ^1^ Otorhinolaryngology Department of Renji Hospital, School of Medicine Shanghai Jiaotong University Shanghai China; ^2^ Shanghai Key Laboratory of Tumor Microenvironment and Inflammation, Department of Biochemistry and Molecular Cell Biology, Institutes of Medical Sciences, School of Medicine Shanghai Jiao Tong University Shanghai China; ^3^ Anesthesia Department of Shanghai International Medical Center Shanghai China

**Keywords:** CRSwNP, macrophage, SENP3

## Abstract

**Background and Aim:**

Small ubiquitin–like modifier (SUMO)‐specific protease (SENP)3 is a protease molecule that responds to reactive oxygen species (ROS) with high sensitivity. However, the role of ROS and SENP3 in the formation of nasal polyps (NPs) remains unclear. This study aimed to explore how SENP3 influenced the outcome of chronic rhinosinusitis (CRS) by altering macrophage function, that is, the formation of NPs.

**Methods:**

The alternative activation of macrophage (M2) was detected with CD68^+^CD206^+^ in humans and CD206^+^ in mice. The nasal mucosa of patients with CRS was tested using flow cytometry (CD68, CD80, and CD206) and triple‐color immunofluorescence staining (CD68, CD206, and SENP3). The bone marrow–derived macrophages from SENP3 knockout and control mice were stimulated with interleukin (IL)‐4 and IL‐13 to analyze alternative macrophage polarization in vitro. An animal model of allergic rhinitis was constructed using SENP3 knockout mice. CD206 was detected by immunofluorescence staining. The thickening of eosinophil‐infiltrated mucosa was detected by Luna staining.

**Results:**

The number of CD68^+^ CD206^+^ M2 increased in the nasal mucosa of patients with CRS with NP (CRSwNP) compared with patients with CRS without NP (CRSsNP), but with no significant difference between the groups. SENP3 knockout increased the polarization of F4/80^+^CD206^+^M2. Meanwhile, the number of CD206^+^M2 significantly increased in the allergic rhinitis model constructed using SENP3 knockout mice and controls, with a more obvious proliferation of the nasal mucosa.

**Conclusion:**

Downregulation of SENP3 promotes the formation of nasal polyps mediated by increasing alternative activated macrophage in nasal mucosal inflammation.

## Graphical abstract

The downregulation of the expression of macrophage specific protease in the nasal mucosa in nasal mucosal inflammation promoted the formation of nasal polyps.

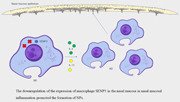

## 1 INTRODUCTION

Nasal polyps (NPs) are proliferating tissue clusters that grow in the mucosa of the nasal cavity or sinus and protrude from the surface of the nasal mucosa. Their clinical symptoms include nasal obstruction or increased nasal secretions, with facial pain or swelling, and hyposmia or anosmia, which often seriously impact the quality of life.^1^ Statistics have shown that about 4% of the general population suffers from NP, and the recurrence rate after nasal polypectomy alone is about 15%–40%.^2^ The pathological features of NP are hypertrophy and extreme edema. It is characterized by epithelial cell shedding and metaplasia, goblet cell metaplasia, connective tissue loosening, mucous gland hyperplasia, and fibrous tissue hyperplasia.^3^, ^4^ The multifactorial effects on the edema and fibrosis of nasal mucosal tissue and tissue remodeling result in irreversible changes in polypoid tissues. A number of studies revealed that the formation of NPs was due to the increased expression levels of Transforming Growth Factor‐β (TGF‐β),^5^ Vascular Endothelial Growth Factor (VEGF),^6^ Platelet Derived Growth Factor (PDGF),^7^ Arginase 1 (Arg‐1),^8^ and other cytokines in local tissues, leading to the excessive proliferation of fibroblasts, accumulation of extracellular matrix, and aggravation of tissue remodeling. On the contrary, the vascular permeability increases under the influence of nasal mucosal inflammation, resulting in edema of the nasal mucosa and accumulation of inflammatory transmitters. In addition, the fibrin stability factor F XIII increases, leading to the aggregation of protein monomers into multimers, further causing the outperformance of fibrosis over anti‐fibrosis of the tissues.^9^ The influencing factors mentioned earlier are closely associated with the alternative activation macrophages (M2).

In inflammatory response, macrophages are the key link in response to inflammatory factors and mediators to the immune system. When the tissues are stimulated by inflammation in nasal mucosal inflammation, the monocytes in the blood rapidly accumulate in local tissues and differentiate into macrophages to activate the immune response and secrete corresponding inflammatory factors.^10^ Under the action of different chemokines, the monocyte–macrophages can be activated into two opposite extreme phenotypes: classically activated macrophage (M1) and alternative activated macrophage (M2). M1 is induced by interferon γ or lipopolysaccharide (LPS). It highly expresses tumor necrosis factor‐α, interleukin 6 (IL‐6), inducible nitric oxide synthase, and other cytokines and is involved in immune responses, such as insulin resistance and killing intracellular parasites and tumor cells. M2 is induced by IL‐4, IL‐13, and glucocorticoids. It can highly express mannose receptor (CD206), arginase 1 (Arg‐1), inflammatory zone molecules (found in inflammatory zone 1), and other cytokines. It is involved in immune responses, such as responding to T Helper Cell Type 2 (Th2) immune response, promoting tissue repair, and inhibiting tumor growth.^11^, ^12^, ^13^ A large number of recent studies have confirmed that the imbalance of M1/M2 exerts an impact on the development and outcome of various diseases, such as pulmonary fibrosis,^14^ cerebral aneurysm,^15^ and breast cancer progression.^16^ Many factors can influence macrophage activation. Small ubiquitin–like modifier (SUMO)‐specific protease (SENP)3 knockout has already been demonstrated to cause macrophage activation with LPS^17^ However, how SENP3 influences macrophage activation under the stimulation of IL‐4 and IL‐13 is still unclear. Moreover, the role of SENP3 and polarization of macrophages in the process of nasal mucosal inflammation and formation of NPs is also unclear.

The SENP family has six members, of which SENP3 is a member located in the nucleolus. It dissociates the SUMO2/3 that is covalently linked to the substrate protein. SUMOylation is a posttranslational modification of proteins, in which SUMO is coupled to the lysine residue in a target protein via an enzymatic pathway.^18^ SUMOylation and deSUMOylation are in a dynamic equilibrium. Under certain conditions, the changes in SENP can cause proteins to have a tendency to undergo SUMOylation and deSUMOylation, which has an impact on the stability of proteins and the activity of other cells, thus changing the development and outcome of the disease. The SUMO family consists of four members, of which only SUMO‐1, SUMO‐2, and SUMO‐3 are present in mammals. SUMO‐2 and SUMO‐3 have a sequence homology of 95%, and the target protein modified by both of them can be specifically removed by SENP3 in the SENP family.^19^ In recent years, the downregulation of the expression of SENP1 may increase M2 polarization.^20^ SENP1 promotes the removal of SUMO‐1 much more efficiently compared with SUMO‐2 and SUMO‐3. It can specifically eliminate SUMO2/3 modification. Therefore, SENP3 was selected in this study to explore the effect of SUMO2/3 on macrophage polarization and its role in the formation of NPs caused by mucosal inflammation in the nasal cavity and sinus. In addition, a large number of reports are available on the high expression levels of SENP3 in tumor cells, such as head and neck squamous cell carcinoma and muscle cachexia.^21^, ^22^ However, studies on the expression of SENP3 in nasal and sinus mucosal inflammation are lacking. Therefore, this study explored how SENP3 affected the outcome of nasal mucosal inflammation by altering macrophage function, that is, the formation of NPs.

## 2 MATERIALS AND METHODS

### 2.1 Study participants

Patients admitted to the Otorhinolaryngology Department, Renji Hospital, Shanghai Jiaotong University School of Medicine, were selected. This study was approved by the ethics committee of Renji Hospital (license number: KY2019‐173). All participants signed the informed consent form before enrolment. A total of 41 patients (Table 1) who underwent nasal mucosal inflammation surgery were included. The diagnosis was based on endoscopic and computed tomography scores and in accordance with the guidelines and consensus for the diagnosis and treatment (Chinese guidelines for diagnosis and treatment of chronic rhinosinusitis. 2018) of nasal mucosal inflammation. These patients were divided into nasal mucosal inflammation with NP (*N* = 28) and nasal mucosal inflammation without NP (*N* = 13) groups, exhibiting nonoverlapping grouping. A total of 15 patients (with NP:without NP = 10:5) underwent immunofluorescence staining and 26 patients (with NP:without NP = 18:8) underwent flow cytometry. The nasal mucosa was harvested from the nasal mucosa adjacent to NPs and the uniform mucosa of patients without NP during the surgery of functional endoscopic sinus. All patients with asthma and aspirin intolerance triad were excluded according to their medical histories and were not treated with antibiotics or glucocorticoids 4 months before the surgery.

**Table 1:** Age and sex distribution of all enrolled patients (*N* = 41).

With NP (*N* = 28)			Without NP (*N* = 13)		
**Sex**		**Age (mean** ± **SD)**	**Sex**		**Age (mean** ± **SD)**
F (*N* = 6)	M (*N* = 22)	49.607 ± 17.240	F (*N* = 6)	M (*N* = 7)	49.077 ± 14.483

### 2.2 Study animals

For this study, 8‐week‐old male C57 BL/6 mice from the same litter, weighing 18–25 g, with a genotype of *Senp3* fl/fl and *Senp3* cKO were selected. The SENP3 knockout mice were obtained by mating *Senp3* fl/fl mice with *Senp3* cKO mice. The control group consisted of mice that were littermates of *Senp3* fl/fl mice. The gene knockout methods and sources were the same as those described in published studies.^17^ All experimental mice were raised in the specific‐pathogen free (SPF) animal room of Shanghai Jiaotong University School of Medicine, with free access to food and water. All animal experiments were performed in strict accordance with the guidelines for the care and use of experimental animals issued by the Ministry of Science and Technology of the People's Republic of China (Guidelines on the Humane Treatment of Laboratory Animals.2006). The program was approved by the Animal Protection and Use System Committee of Shanghai Jiaotong University School of Medicine (license number: A‐2019‐041). After continuous administration of nasal drops for 12 weeks, the mice were sacrificed 30 min after the last nasal cavity stimulation. Then, the hearts were rinsed with 20 mL of phosphate‐buffered saline (PBS) and fixed with 20 mL of 4% paraformaldehyde. After the fixation, the nasal specimens were harvested according to the procedures described by Dunston et al.^23^ The scalp was peeled, the zygoma and mandible were cut, and the nose was dissected from the posterior part of the eyelid. The primary bone marrow derived macrophages (BMDMs) were extracted and induced in the same manner as described by Weischenfeldt et al.,^24^ with some changes in terms of the incubation method. Also, after the mice were sacrificed, the femur and tibia were separated, the muscles were removed, and the bilateral metaphyses were opened with a sterile ophthalmoscope. Then, a 1‐mL syringe was punched out from one metaphysis end to the other end with α‐minimum Eagle's medium (α‐MEM; Gibco, 12571‐063), followed by filtering with a 70‐µm MACS SmartStrainer (Miltenyi, 130‐098‐462) and incubation.

### 2.3 Culture of BMDMs and induce polarization of macrophages

The culture method was as described by Weischenfeldt et al.^24^ The cells were extracted from the leg bones of mice with mononuclear macrophage Senp3 flox/flox Lyz2‐cre (Senp3 cKO) and mice with Senp3 flox/flox (Senp3 fl/fl), and then incubated with α‐MEM, 10% fetal bovine serum (Hyclone, sv30087.03), and 50 ng/mL macrophage colony‐stimulating factor (M‐CSF) (Sino Biological, 511112‐MNAHa) for 7 days. Then followed by F4/80 staining for flow cytometry. The black arrow indicated macrophages (M0), which was round and unpolarized (Supporting Information: Figure S1A and Figure 4B). The results showed that more than 99% of bone marrow–derived monocytes became M0 after induction in vitro (Supporting Information: Figure S1C and Figure 4D). Then the successfully induced macrophages was divide equally in a six‐well plate and co‐stimulated with 20 ng/mL IL‐4 (Sino Biological, 51084‐MNAE) and 10 ng/mL IL‐13 (Sino Biological, 50225‐MNAH) for 48 h to induce M2.

### 2.4 Allergic rhinitis modeling

The model was constructed as described by Kim et al.^25^ and Wang et al.^26^ A model of allergic rhinitis with Th2 immune response was used. The whole process was carried out in the SPF animal room, with no bacteria or fungi colonized in the nasal cavity, to eliminate the uncertain effects of bacteria or fungi on macrophages and NPs. The model was constructed using four *Senp3* fl/fl mice and five *Senp3* cKO mice.

Sensitization phase: The animals were intraperitoneally injected with 100 µL of chicken ovalbumin (OVA) (Sigma, A5503‐1g) dissolved in PBS (75 µg/mouse) and Imject Alum Adjuvant (Thermo Fisher Scientific, 77161, USA; 50 µL/mouse), once every alternate day for seven times.

Stimulating phase: The mice were dripped with 25 µg/µL OVA, once a day, for 12 weeks, 10 µL per nostril, and inverted for 5 min after administering nasal drops to prevent OVA from entering the airway. The symptoms of the mice were observed 10 min after each stimulation. Nine mice showed behaviors of scratching the nose and ears and sneezing. The symptom scores met the criteria for the allergic rhinitis model.^25^, ^26^ Luna staining revealed eosinophil infiltration.

### 2.5 Immunofluorescence staining

Macrophages and SENP3 protein obtained from the nasal mucosa of patients were subjected to immunofluorescence co‐localization staining, while the macrophages in the nasal specimens of mice with allergic rhinitis were subjected to immunofluorescence staining. The specimens were fixed with 4% paraformaldehyde, embedded in paraffin, and then separately incubated with CD68 antibody 1:2000 (Service, GB13067‐M‐2), CD206 antibody 1:200 (Service, GB11062), and SENP3 antibody 1:5000 (5591S, CST) overnight at 4°C in the dark. Subsequently, the specimens were incubated with horseradish peroxidase–labeled goat anti‐mouse or anti‐rabbit antibody 1:500 (Service, GB2330) at room temperature for 50 min and then incubated with Future Innovation Technology Creativity (FITC), CY3, and 1:200 594 goat anti‐rabbit fluorescence secondary antibody (Service) at room temperature in the dark for 10 min, followed by counterstaining with 4',6‐diamidino‐2‐phenylindole (DAPI; Service, G1012) and observation under a microscope. The wavelength of DAPI ultraviolet light was 330–380 nm (blue light), FITC was 465–495 nm (green light), CY3 was 510–560 nm (red light), and 594 was 594 nm (rose red). The immunofluorescence staining of nasal specimens obtained from mice followed a slightly different procedure. After fixation with 4% paraformaldehyde, the specimens were decalcified with 10% ethylenediaminetetraacetic acid for 2 weeks, followed by paraffin embedding. The immunofluorescence staining was performed using CD206 (Service, GB11062). An upright fluorescence microscope (Nikon Eclipse C1) and imaging system (Nikon DS‐U3) were used for observation.

### 2.6 Luna staining

The Luna staining procedure referred to the method described by Van de Rijn M et al.^27^ The cells were stained for 5 min using the mixture of Weiger's iron hematoxylin (0.005% hematoxylin and 0.6% ferric chloride incubated in 2% hydrochloric acid) and Biebrich scarlet solution (Biebrich scarlet and 0.1% acid fuchsin incubated in 1% acetic acid for 5 min) in a ratio of 9:1. They were then differentiated with 1% hydrochloric ethanol and soaked in 0.5% lithium carbonate solution for 1 min, followed by washing, dehydration, mounting, and observation under a light microscope. The cells specifically stained scarlet were eosinophils.

### 2.7 Flow cytology

#### 2.7.1 Digestion of the nasal mucosa of patients before testing

The digestive fluid was formulated using 250 U DNaseI (Sigma, D5025‐15KU), 5‐mg type 2 collagenase (Sigma, C6885‐100MG), and 2.2 mL of Dulbecco's Modified Eagle Medium (DMEM) (Gibco, C11960500BT). After filtration and washing, the suspension was incubated with 1:1000 Fixable Viability Stain 510 (BD Pharmingen, 564406) at room temperature in the dark for 15 min, mixed with 1:50 Fc Block (BD Pharmingen, 564219), and sealed at 4°C for 5 min. Further, it was mixed with CD11b antibody 1:100 (BD Pharmingen, 557396A) and CD80 antibody 1:100 (BD Pharmingen, 557227) and incubated at room temperature in the dark for 30 min, followed by membrane rupture using a membrane rupture kit (BD Pharmingen, 554714) at 4°C for 20 min. Then, it was incubated with CD68 antibody 1:100 (BD Pharmingen, 564943) and CD206 antibody 1:100 (BD Pharmingen, 550889) at room temperature in the presence of light for 30 min, washed, and tested with Beckman CytoFlex flow cytometry.

#### 2.7.2 Quantitative analysis of BMDMs by flow cytometry

After the cultured BMDM controls were seeded in a six‐well plate, the cells were carefully scraped off using a cell scraper. After washing, the suspension was sealed with 1:50 Fc Block (BD Pharmingen, 553141) at 4°C for 5 min and incubated with F4/80 antibody 1:100 (eBioscience, 48‐4801‐82) at room temperature in the dark for 30 min, followed by membrane rupture using a membrane rupture kit (BD Pharmingen, 554714) at 4°C for 20 min. Further, it was incubated with CD206 (BD Pharmingen, 141710) at room temperature in the light for 30 min, washed, and tested using a Beckman CytoFlex flow cytometer.

### 2.8 Western blot analysis

The cultured BMDM controls were seeded in a six‐well plate. The protein was extracted with 150 µL of 4× sodium dodecyl sulfate per well and quantified using a NanoDrop2000 spectrophotometer. The gel was prepared using a 10% polyacrylamide gel electrophoresis gel kit (PG112, EpiZyme). Each well was loaded with 5–10 µL of the gel, and 5 µL of prestained protein ladder (Thermo Fisher Scientific, 00594048) was added on both sides, followed by electrophoresis at a constant voltage of 90 V for 90 min. The suspension was sealed with 5% skimmed milk powder and then incubated with SENP3 antibody 1:1000 (CST, 5591S) and β‐actin antibody 1:1000 (Sigma, MAB8929) overnight at 4°C. Subsequently, 5% skimmed milk powder was separately added to dissolve SENP3 antibody 1:5000 (Jackson ImmunoResearch, 111‐035‐003, USA) and anti‐β‐actin antibody 1:5000 (Jackson ImmunoResearch, 115‐035‐003). The β‐actin was developed using electrochemiluminescence colored solution (Pierce, 34580, USA), and SENP3 was developed using an ultra‐sensitive colored solution (Merck, wbkls0500).

### 2.9 Statistical analysis

Data were analyzed using SPSS 23 software. Data from flow cytometry were acquired and analyzed using CytExpert. Statistical analyses were performed using *t* tests.

## 3 RESULTS

### 3.1 Increased M2 polarization phenotype in the nasal mucosa

**Figure 1:**
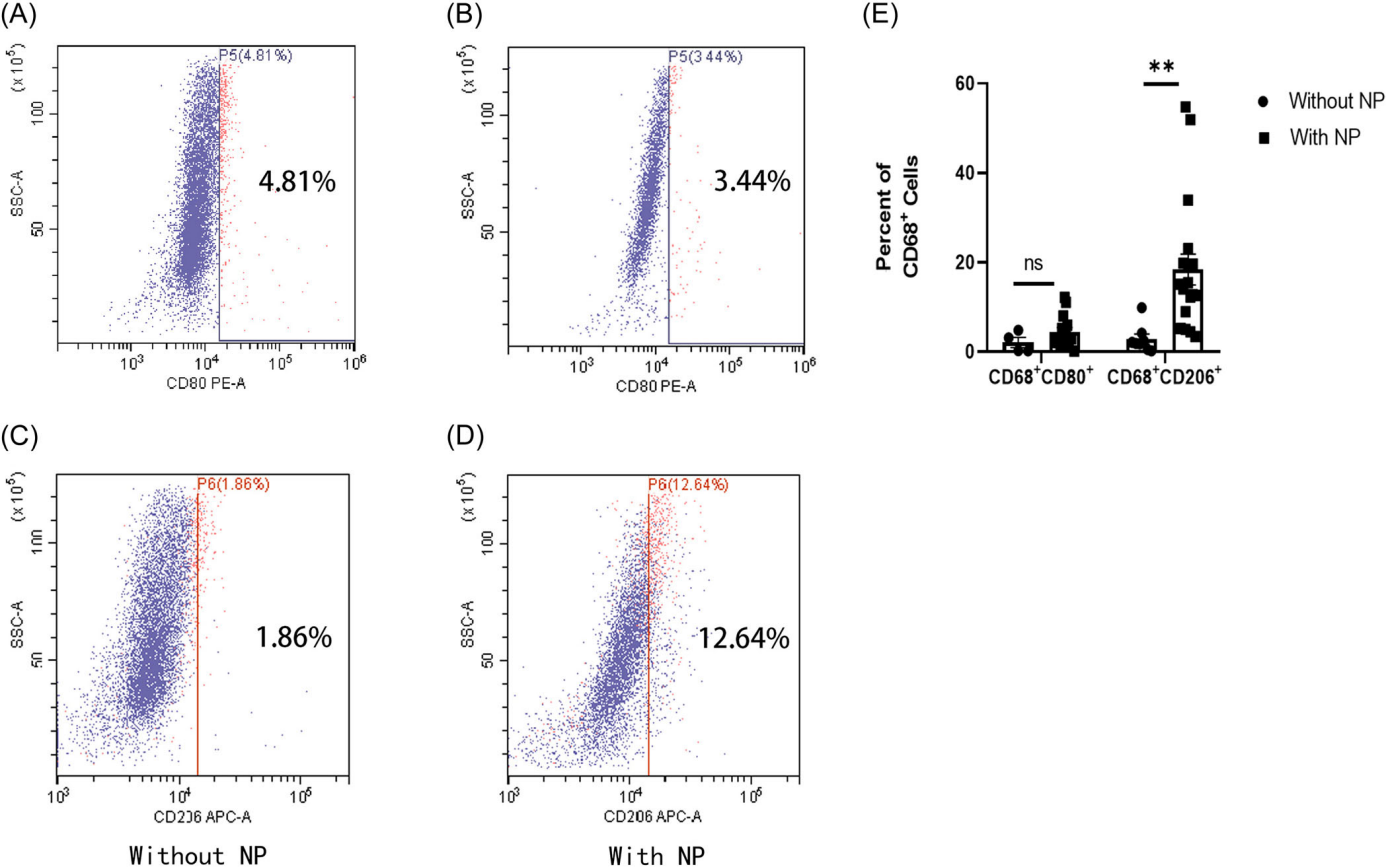
M2 subtype increased in the nasal mucosa of patients accompanied by the formation of nasal polyps (NPs), as detected by flow cytometry. Representative cell spots of the nasal mucosal inflammation without NP group (A, C) and nasal mucosal inflammation without NP group (B, D). Flow cytometric analysis of macrophages in the nasal mucosa by classical activation (M1) and alternative activation (M2). Flow cytometry analysis (CD80 [PE‐A] and CD206 [APC]) was gated by Fixable Viability Stain510 (KO525), CD11b (FITC), and CD68 (PB‐450), revealing that the number of M2 (FV510^+^CD11b^+^CD68^+^CD206^+^) significantly increased in the nasal mucosa of the nasal mucosal inflammation with NP group compared with the nasal mucosal inflammation without NP group (*p* = .0076), but with no significant difference in the number of M1 macrophages (FV510^+^CD11b^+^CD68^+^CD80^+^) between the two groups (*p* = .2553) (E). Positive cells were counted, and the data were expressed as mean ± standard error of mean for each group. The statistical analysis was performed using the multiple *t* test **p < .01.

The nasal mucosa harvested during the surgery was analyzed using flow cytometry between the nasal mucosal inflammation with NP (*N* = 18) and nasal mucosal inflammation without NP (*N* = 8) groups (Figure 1E). The results revealed no significant difference in the number of M1 (FV510^+^CD11b^+^CD68^+^CD80^+^) (*p* = .2553) (Figure 1A,B), but the number of M2 (FV510^+^CD11b^+^CD68^+^CD206^+^) was significantly higher in the nasal mucosal inflammation with NP group than in the nasal mucosal inflammation without NP group (18.415 ± 3.484 vs. 2.876 ± 1.085, *p* = .0076) (Figure 1C,D). Positive cells were counted, and the data were expressed as mean ± standard error of mean for each group. Statistical analysis was performed using the multiple *t* test, **p < .01.

### 3.2 SENP3 loss increased the number of M2 macrophages in the nasal mucosa

**Figure 2:**
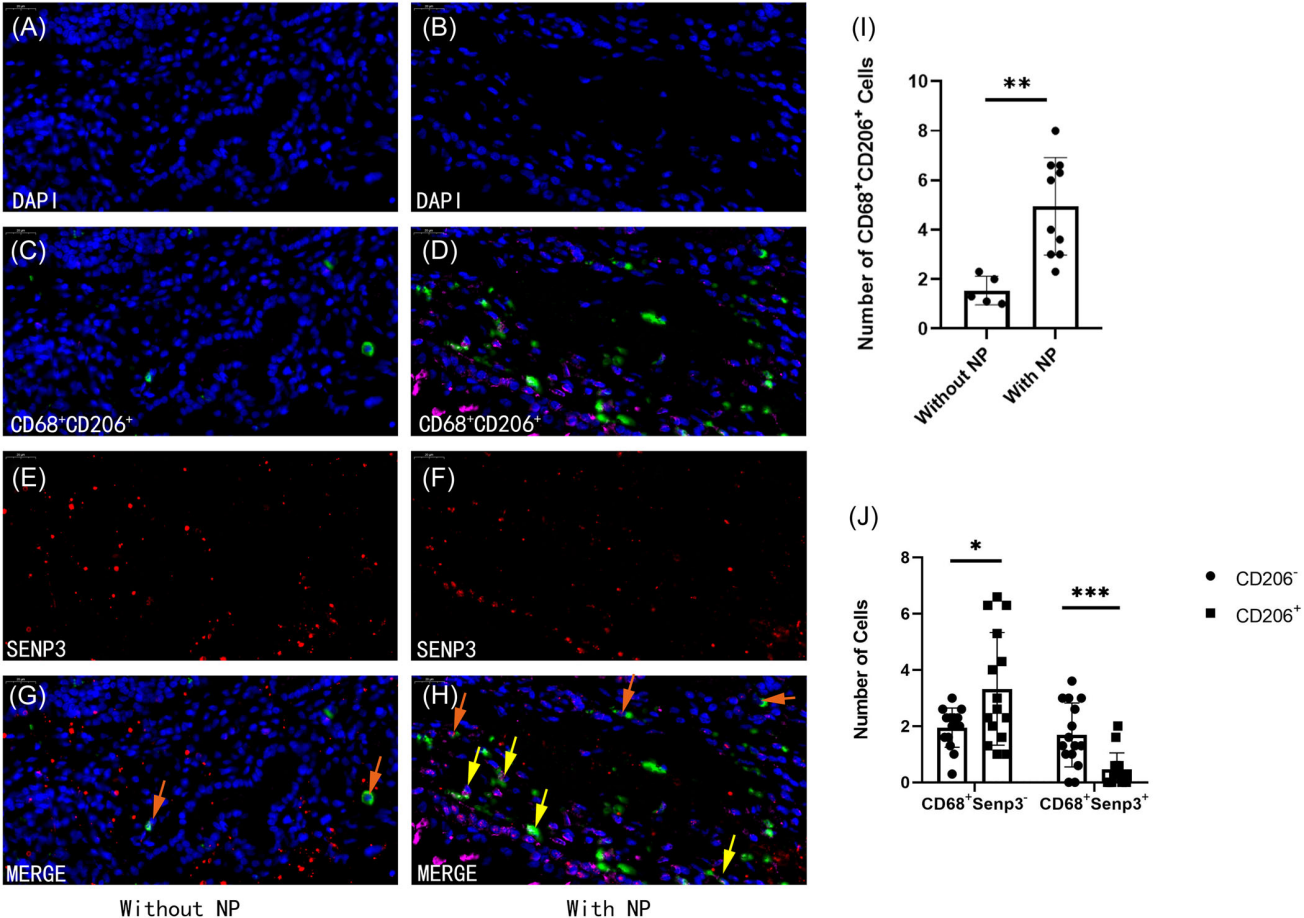
Immunofluorescence staining of paraffin sections of nasal mucosa intraoperatively collected from patients with rhinitis. On comparing the nasal mucosal inflammation without NP group (A, C, E, and G) with the nasal mucosal inflammation with NP group (B, D, F, and H), same as the flow cytometry，the number of CD68^+^CD206^+^ cells was found to be increased in the nasal mucosal inflammation with NP group (C, D, I) (*p* = .0026). At the same time, the number of CD68^+^SENP3^−^ cells without the expression of CD206 was less than those with the expression of CD206 (*p* = .0176) and the number of CD68^+^SENP3^+^ cells without the expression of CD206 was more than those with the expression of CD206 (*p* = .0009) (G, H, J). The orange arrow indicates CD68^+^SENP3^+^CD206^−^cells, and the yellow arrow indicates CD68^+^SENP3^−^CD206^+^ cells. The cells were counted by three different technicians under random fields. Positive cells were counted, and the data were expressed as mean ± standard error of mean for each group. The statistical analysis was performed using the *t* test *p < .05, **p < .01, ***p < .001. The scale in the figure is 20 μm.

The nasal mucosa harvested during the surgery was subjected to immunofluorescence staining to investigate the effects of SENP3 on the recruitment and polarization phenotype of macrophages (Figure 2). Similar to flow cytometry, the results showed that the M2 macrophages were significantly more in the nasal mucosal inflammation with NP group (*N* = 10) than in the nasal mucosal inflammation without NP (*N* = 5) group (4.940 ± 1.973 vs. 1.540 ± 0.577, *p* = .0026; Figure 2C,D, and 2I). Meanwhile, the number of CD68^+^SENP3^−^ cells without the expression of CD206 was less than the number of cells with the expression of CD206 (1.947 ± 0.694 vs. 3.327 ± 2.002; *N* = 15, *p* = .0176), and the number of CD68^+^SENP3^+^ cells without the expression of CD206 was more than the number of cells with the expression of CD206 (1.687 ± 1.135 vs. 0.460 ± 0.593; *N* = 15, *p* = .0009; Figure 2G,H, and 2J), suggesting that SENP3 might be involved in M2 polarization (the orange arrow indicating CD68^+^SENP3^+^CD206^−^cells, and the yellow arrow indicating CD68^+^SENP3^−^CD206^+^ cells). Positive cells were counted, and the data were expressed as mean values of this mean ± standard error of mean for each group. Statistical analysis was performed using the *t* test, *p < .05, **p < .01, and ***p < .001.

### 3.3 Eosinophilic nasal mucosal inflammation may promote the formation of nasal polyps and smoking didn't promote nasal polyp formation by increasing M2/M

**Figure 3:**
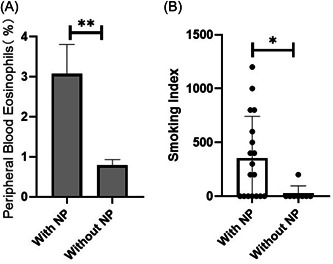
Patients with nasal polyps had higher levels of peripheral blood eosinophils than patients without nasal polyps (*p* = .0088) (A). Smoking Index of patients with nasal polyps was significantly higher than that of patients without nasal polyps (*p* = .0260) (B). The data were expressed as mean values of this mean ± standard error of mean for each group. Statistical analysis was performed using the paired *t* test, *p < .05, **p < .01.

We analyzed the furnishing demographic and clinical characteristics of 26 patients analyzed by flow cytometry and obtained the following results. Patients with nasal polyps had higher levels of peripheral blood eosinophils than patients without nasal polyps (*N* = 26, *p* = .0088; Figure 3A). Age and sex had no effect on M2/M and peripheral blood eosinophils (Figure S2A–C). There was also no significant difference in age distribution between patients with and without nasal polyps (Figure S2D). In the survey of the smoking status of the sample, we found that the smoking index of patients with nasal polyps was significantly higher than that of patients without nasal polyps (*N* = 26, *p* = .0260; Figure 3B). However, smoking had no significant effect on M2/M and peripheral blood eosinophils (Figure S2E,F). The data were expressed as mean values of this mean ± standard error of mean for each group. Statistical analysis was performed using the paired *t* test, *p < .05, **p < .01.

### 3.4 Increased proportion of alternatively activated bone marrow–derived macrophages in *Senp3* knockout mice in vitro

**Figure 4:**
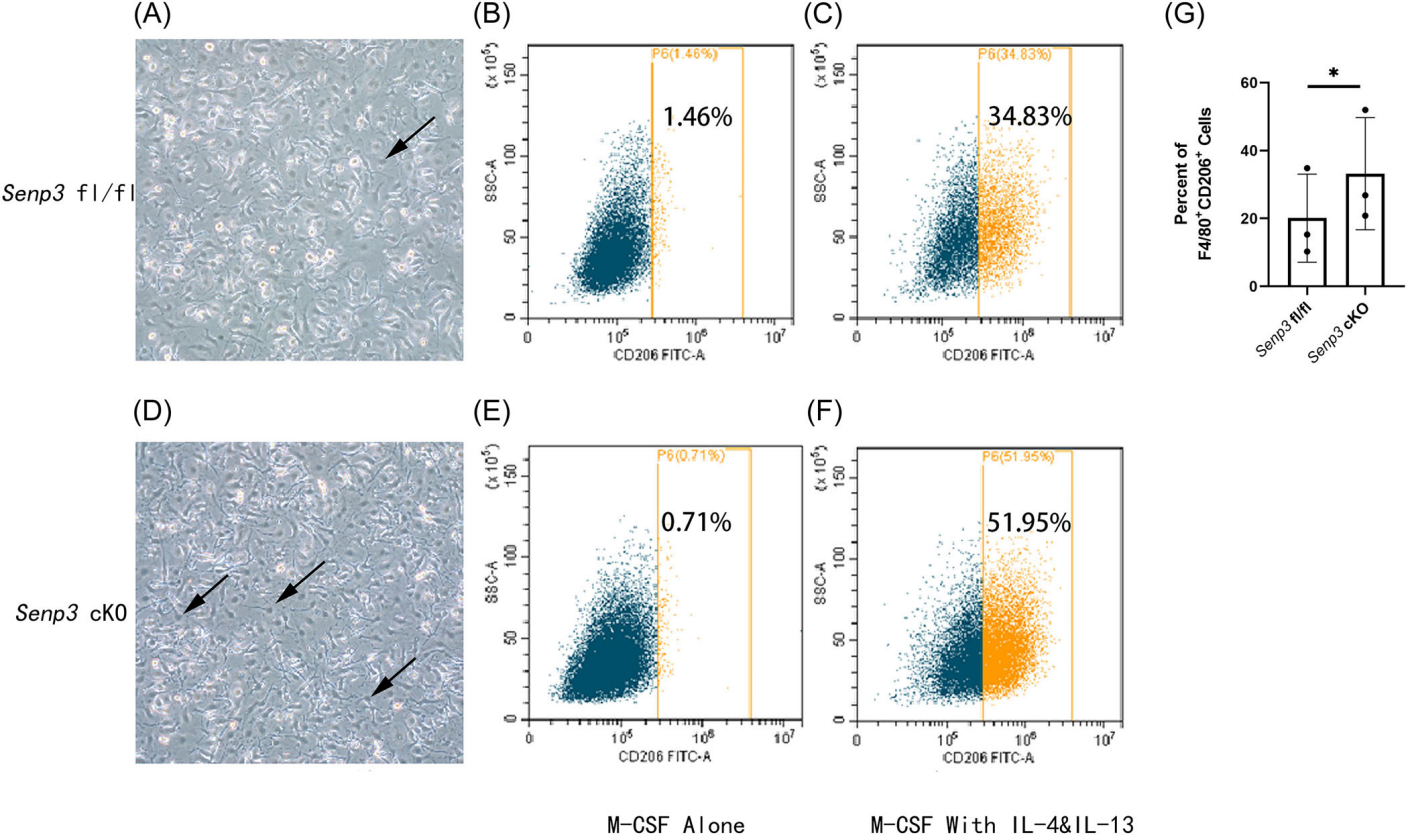
Induced macrophages were stimulated with IL‐4 and IL‐13 and observed under a microscope (A, D). After stimulation with IL‐4 and IL‐13 for 48 h, the cells in the *Senp3* fl/fl group (B, C) and the *Senp3* cKO group (E, F) were analyzed using flow cytometry. After treatment with F4/80 and single‐staining with CD206（FITC), BMDM in the *Senp3* cKO group promoted macrophage polarization to M2, with an increase of about 49%. The in vitro experiment was repeated another two times, which showed basically the same proportion of macrophage polarization to M2 in the *Senp3* cKO group (G) (*p* = .0235). The statistical analysis was performed using the paired *t* test *p < .05. For each sample more than 50,000 events were recorded.

Microscopically, the black arrow indicated M2, which was round with short stubby pseudopodium in *Senp3* fl/fl and *Senp3* cKO mice (Figure 4A,D). The quantitative analysis by flow cytometry revealed an increase in the proportion of macrophages polarized to M2 (Figure 4B,C,E,F). These results indicated that *Senp3* cKO promoted the polarization of monotype‐derived macrophages to M2 (*p* = .0235; Figure 4G). Statistical analysis was performed using the paired *t* test, *p < .05. For each sample more than 50,000 events were recorded.

### 3.5 Lower expression level of SENP3 in M2 polarization with the stimulation of IL‐4 and IL‐13

**Figure 5:**
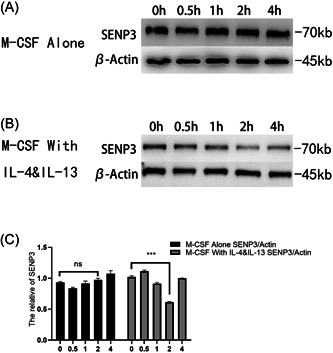
BMDM was extracted from the leg bones of *Senp3* fl/fl and stimulated with IL‐4 and IL‐13 in a time gradient of 0, 0.5, 1, 2, and 4 h. The SENP3 expression was evaluated using the Western blot semi‐quantitative analysis. The results showed that M‐CSF hardly affected the expression of SENP3 after the maturation and stabilization of macrophages (A), but the SENP3 expression level decreased after stimulated with IL‐4 and IL‐13 (B). The expression of SENP3 decreased most significantly at 2 h of induction (C) (*p* = .000005). The statistical analysis was performed using the *t* test ***p < .001.

Although M2 polarization increased in the pathological state of *Senp3* cKO mice in vitro, SENP3 was not completely knocked out under physiological conditions. The role of SENP3 in M2 polarization remained unknown. Therefore, BMDMs extracted from the leg bones of *Senp3* fl/fl mice, was incubated, induced, and then stimulated with IL‐4 and IL‐13 for 0, 0.5, 1, 2, and 4 h. The expression level of SENP3 in BMDM cells 0, 0.5, 1, 2, and 4 h after the medium was changed was evaluated 7 days later to exclude the effect of M‐CSF, which was needed to induce macrophages. M‐CSF hardly affected the expression of SENP3 after the maturation and stabilization of macrophages (Figure 5A). After stimulation with IL‐4 and IL‐13, the expression level of SENP3 significantly decreased in 0.5–4 h (Figure 5B). The expression of SENP3 decreased most significantly at 2 h of induction (Figure 5C) (*p* = .000005). The statistical analysis was performed using the *t* test ***p < .001.

**Figure 6:**
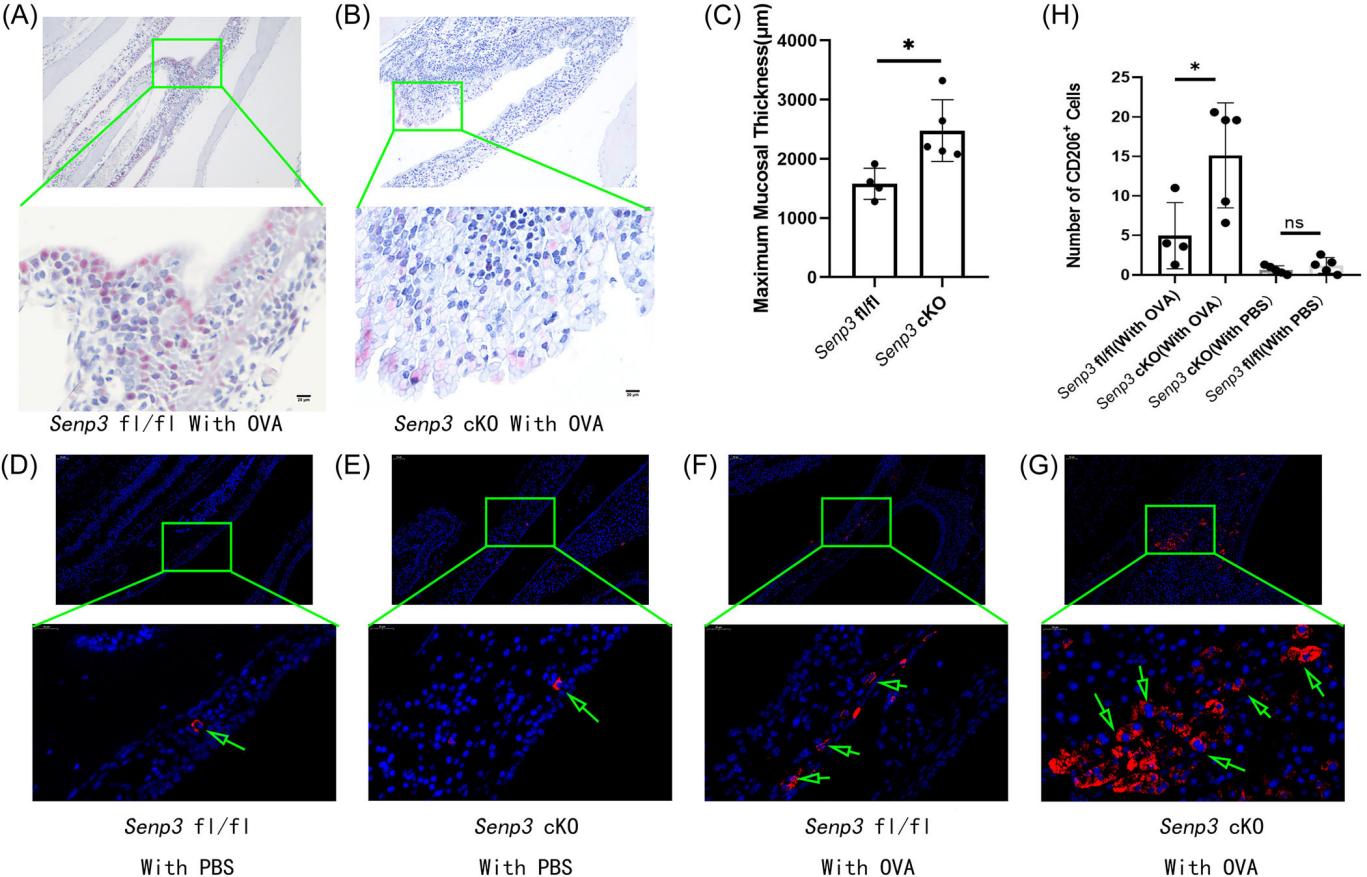
The specimens were fixed, decalcified, paraffin‐embedded, serially sectioned, and subjected to Luna eosinophil–specific staining (A, B) and immunofluorescence staining with CD206 (D, E, F, and G). Luna staining suggests that the mucosa infiltrated by eosinophils in the *Senp3* cKO group was thicker than that in the *Senp3* fl/fl group (C,*p* = .0172). Immunofluorescence staining shows that the number of CD206^+^ cells in the group with OVA was more than that in the group with PBS. Otherwise, the number of CD206^+^ cells in the *Senp3* cKO group was more than that in the *Senp3* fl/fl group (*p* = .0331) (D, E, F, and H). CD206^+^ cells were counted by the mean of three different technicians under random fields. The data are expressed as mean ± standard error of mean for each group. The statistical analysis was performed using the *t* test *p < .05. The scale in the figure is 20 μm.

### 3.6 *Senp3* cKO increased the number of M2 and caused mucosal thickening in animal models of allergic rhinitis

Models of allergic rhinitis were constructed using *Senp3* cKO mice (*N* = 5) and *Senp3* fl/fl mice (*N* = 4) to further confirm the hypothesis that *Senp3* cKO promoted the formation of NPs by affecting M2 polarization. The mice in this model were given continuous OVA suspension drops. Moreover, *Senp3* cKO mice (*N* = 5) and *Senp3* fl/fl mice (*N* = 5) were given PBS. The mice were continuously given nasal drops for 12 weeks according to the diagnosis of nasal mucosal inflammation. Luna eosinophil–specific staining suggested that the mucosa infiltrated by eosinophils in the *Senp3* cKO group was thicker than that in the *Senp3* fl/fl group (2476.584 ± 522.458 vs. 1578.415 ± 264.650; *p* = .0172; Figure 6A–C). Immunofluorescence staining showed that the number of CD206^+^ cells in the group with OVA was more than that in the group with PBS (Figure 6D,H). Otherwise, the number of CD206^+^ cells in the *Senp3* cKO group was more than that in the *Senp3* fl/fl group (15.140 ± 6.645 vs. 4.975 ± 4.189; *p* = .0331; Figure 6F–H). The data were expressed as mean values of this mean ± standard error of mean for each group. Statistical analysis was performed using the *t* test, *p < .05.

## 4 DISCUSSION

Overall, this study has certain limitations. From the perspective of subjects, SENP3 could not be completely knocked out in human nasal mucosa as in experimental animals. Therefore, there may be some bias in the conclusion of the experiment. From the experimental results, there is no clear definition of nasal polyp formation in the literature. We looked for evidence of nasal polyp formation based on the pathophysiological characteristic of nasal polyp formation. Therefore, the validity of the results will increase as the sample size increased.

In the experiment, we used paranasal nasal polyp mucosal tissue, bone marrow‐derived macrophages, and nasal mucosal tissue of the AR mouse model. Though a large number of studies showing that inflammation of the nasal mucosa with eosinophil's infiltration promotes the formation of nasal polyps,^28^, ^29^ we observed that the peripheral blood eosinophil count was entirely random in the patiernts with NPs. It is suggested that eosinophils infiltration is not a decisive factor in the formation of nasal polyp. Although the formation mechanism of nasal polyps is uncertain, tissue remodeling of nasal mucosa under inflammatory stimulation is widely reported.^30^, ^31^ In these reports, TGF‐β is considered to be the key of the nasal mucosa tissue remodeling cytokines, the main source of which is alternative activated macrophages (M2) in nasal polyps. We also observed alternative activated macrophages (M2) increased in the inflamed nasal mucosa. It suggests that alternative activated macrophages mediated inflammation may be an important process of nasal mucosa tissue remodeling. Mouse bone marrow‐derived macrophages were used to verify the effect of SENP3 on the polarization of alternative activated macrophages (M2). Nasal mucosa of allergic rhinitis model mice was used to verify the influence of SENP3 on the alternative activated macrophages (M2) polarization under nasal mucosa inflammation and the correlation between alternative activated macrophages (M2) polarization and the process of nasal mucosal tissue remodeling.

In the present study, the M2 phenotypes in the nasal mucosa collected from patients suffering from nasal mucosal inflammation with NP and nasal mucosal inflammation without NP were subjected to immunofluorescence staining. The number of M2 in the inflammatory mucosa collected from patients with nasal mucosal inflammation with NP significantly increased compared with that from patients with nasal mucosal inflammation without NP, which was characterized by the increased number of CD68^+^CD206^+^ cells (Supporting Information: Figure S3). Similarly, the flow cytometry analysis of specimens revealed that the number of M2 in the nasal mucosa collected from patients with nasal mucosal inflammation with NP was significantly higher than that from patients with nasal mucosal inflammation without NP, which was consistent with the data reported in published studies.^28^ These results indicated that the formation of NP was closely associated with tissue remodeling caused by M2 polarization. In the progression of nasal mucosal inflammation, *Staphylococcus aureus* infection prompted the polarization of macrophages into M2 and the formation of NPs.^32^ Obviously, for macrophages in patients with nasal and sinus inflammation, complicated factors affected M2 polarization, *S. aureus* infection being only one of them. However, other factors were not addressed. A large number of studies confirmed that for patients with airway mucosal inflammation, smoking was a key factor in the recurrence of sinusitis and the formation of NPs.^33^, ^34^ Moreover, the number of reactive oxygen species (ROS) produced by the nasal mucosa in smoking patients was significantly higher than that in nonsmoking patients.^35^, ^36^ Interestingly, SENP3 is continuously degraded by the ubiquitin–proteasome pathway under basal conditions, while ROS inhibits this degradation and leads to a large amount of accumulated SENP3,^37^ which differs from the findings of the present study. This study found that a small number of macrophages simultaneously expressed markers of M1 and M2. Hence, an interconversion relationship was thought to exist between M1 and M2, which was consistent with the findings of Shang et al. showing that adipose‐derived stem cells could promote the conversion of M1 into M2.^38^ According to the previous results,^17^ the knocking out of SENP3 could inhibit LPS‐induced macrophages. It was speculated that ROS produced by tobacco increased the expression level of SENP3, promoted the deSUMOlation of macrophages, and induced the polarization of macrophages to M1. The reaction was reversed with the degradation of ROS. Although the polarization of M1/M2 could be regulated by maintaining the balance of SUMOylation and deSUMOylation, the expression of cytokines by M1/M2 and the resulting phenotypic effects, such as tissue remodeling, were irreversible, which ultimately led to an irreversible outcome of the disease. Whether the SENP3^−^mediated deSUMOylation is a regulator of macrophage polarization needs to be confirmed. However, the findings undoubtedly provided a potential direction for research on diseases affected by M1/M2.

The present study investigated the expression of SENP3 at different time gradients after the BMDMs collected from wild‐type mice were stimulated with IL‐4 and IL‐13. A significant increase in the expression level of SENP3 was observed after 24–48 h, which was inconsistent with the conclusion that knocking out of SENP3 promoted the number of M2. A feedback regulation mechanism might exist for the downregulation of expression of SENP3 to promote M2. In terms of the feedback regulation of the SENP family, Cui et al. found that SENP1 promoted hypoxia‐inducible factor‐1α (HIF‐1α) in hepatoma cells, and HIF‐1α could regulate SENP1 in a positive feedback manner through a hypoxia response element.^39^ However, Cui et al. did not address studies on the feedback regulation of SENP3. In their study, SENP1 was stably expressed in hepatoma cells, while the expression level of SENP3 decreased, suggesting the possibility of negative feedback regulation of SENP3, which was consistent with the hypothesis in the present study. On the contrary, since SUMOylation is a reversible reaction of dynamic equilibrium, IL‐4 and IL‐13 remain in a stable state during the Th2 immune response in the body, which does not decrease over time. However, in in vitro experiments, the expression of SENP3 by macrophages fluctuated relatively as cells consumed IL‐4 and IL‐13. The expression first decreased and then increased, which was the limitation of in vitro experiments. The experiments confirmed that the expression level of macrophage SENP3 decreased in response to Th2 immune reaction. However, the reason for the increase in the expression level of SENP3 in the later stage remains to be explored.

According to Enache et al.^40^ the pathological feature of nasal polyp includes the heterogeneity of epithelial and stromal changes. Stromal changes were represented by edema and inflammatory cells infiltration. In our experiment, we found that after HE staining of the nasal mucosa, both SENP3 fl/fl and SENP3 knockout can be observed with cell edema, inflammatory cell infiltration, basal layer thickening (Supporting Information: Figure S4). B Petruson's study in 1988 found that the inflammatory factors secreted by macrophages in nasal mucosa can stimulate the proliferation of nasal mucosa for a long time.^41^ Similarly, in recent years, a large number of research reported that alternative activated macrophages (M2) can promote the formation of nasal polyp through inducing fibroblast proliferation, increasing vascular permeability, and enhancing coagulation function by secreting TGF‐β.^6^ Therefore, according to the results of HE staining, it suggests that the inflammation promoted by M2 polarization accelerates nasal mucosal hyperplasia, which conforms to the pathophysiological process of nasal polyps.

The present study found that the knocking out of SENP3 had a promoting effect on alternative activated macrophages, suggesting that a SUMO2/3 SUMOylation modification might be involved in alternative activated macrophages, which was consistent with the findings of Wang et al. showing that the SENP1 knockout‐catalyzed KLF4 SUMOylation could promote alternative activated macrophages.^20^ The results of this experiment were of great significance for exploring the posttranslational modification of SUMO2/3 loci as an important transcription factor during the process of alternative activated macrophages. For a long time, M2 was considered an immune response molecule for Th2 immune response.^42^ In the TH2‐type immune response, IL‐4 and IL‐13 generated by Th2 cells and eosinophils were the most direct‐acting cytokines involved in alternative activated macrophages. IL‐4 formed types I and II complexes with IL‐4Rα, while IL‐4Rα formed type II complex by recruiting IL‐13Rα1.^43^, ^44^ Meanwhile, the types I and II complexes stimulated the phosphorylation of signaling transducer and activator of transcription 6 (STAT6) via the Janus kinase pathway.^45^ Thus, the macrophages used alternative activation to respond to TH2‐type immune response. This approach was the most classical mechanism for alternative activated macrophages. In addition, Odegaard et al. found that peroxisome proliferator–activated receptor γ (PPARγ) and PPARδ could enhance alternative activated macrophages by synergizing the response of macrophages to IL‐4 and IL‐13 at the transcriptional level, thereby contributing to hepatic metabolic disorders and type 2 diabetes.^46^, ^47^ Moreover, MacKinnon et al. used small interfering RNA (siRNA)‐targeted galectin‐3, mouse knockout model, and specific galectin‐3 inhibitors to block the expression of the major markers of M2 by inhibiting the phosphatidylinositol 3 kinase pathway, thereby clarifying a key mechanism for the activation of activated macrophages via the IL‐4/IL‐13 pathway.^48^ Although many studies focused on M2, most of them were confined to the transcriptional level. Nonetheless, the effect of ubiquitination of SUMO2/3 on alternative activated macrophage was never reported. The overexpression of SENP3 in 293 T cell lines was found to inhibit STAT6 phosphorylation, which could be reversed after stimulation with IL‐4 and IL‐13 2 h (Supporting Information: Figure S5). In addition, eosinophil infiltration was considered to be one of the characteristics of Th2 immune response disease. Studies revealed that in patients with nasal mucosal inflammation, the number of M2 increased in the nasal mucosa of patients with eosinophilia compared with patients without eosinophilia.^49^ Hence, eosinophils play an important role in the formation of NPs in patients with nasal mucosal We also observed eosinophils increased in the alternative activated macrophages inflammation. Schneider et al.^50^ found that the knocking out of STAT6 could clear eosinophils induced by OVA and infiltrated by airway mucosal inflammation, strongly suggesting that the formation of NPs correlated with M2 polarization through STAT6 transcription factors. Previous studies showed that SENP3 enhanced STAT3 phosphorylation by hydrolyzing STAT3‐bound SUMO2/3 modification.^20^ These findings indicated that STAT6‐decoupled SUMO2/3 modification affected the alternative activated macrophages, thereby promoting the formation of NPs in Th2 immunoreactive nasal mucosal inflammation. There might be two kinds of way on how does the deSUMOylation of SUMO2/3 promote STAT6 phosphorylation, directly and indirectly. There is no literature reporting the exact site about STAT binding with SUMO2/3 directly. Similarly, we tried to use CO‐IP to explore the phosphorylation of STAT6 whether combining SUMO2/3, the results were negative. According to Zhou et al.,^21^ phosphatase TC45 combined with SUMO2/3 accelerate the TC45 combinated with STAT3, which promotes the phosphorylation of STAT3. This suggests that a particular phosphorylase may be the mechanism for promoting STAT6 phosphorylation after low SENP3 expression. This study proposed a new insight for the polarization of M2 and even the formation of NPs from the perspective of the molecular biology of posttranslational modification.

## 5 CONCLUSION

Downregulation of SENP3 promotes the formation of nasal polyps mediated by increasing alternative activated macrophage in nasal mucosal inflammation.

## AUTHOR CONTRIBUTIONS

Zheng Zhou conceived the study and participated in writing the manuscript. Ximing Bao performed the experiments, analyzed the data, and wrote the manuscript. Jiping Li and Jie Yang participated in writing the manuscript. All authors read and approved the final version of the manuscript.

## CONFLICT OF INTEREST STATEMENT

The authors declare no conflicts of interest.

## ETHICS STATEMENT

Shanghai Jiaotong University School of Medicine, Renji Hospital Ethics Committee Approval Letter (license number: KY2019‐173). Animal Protection and Use System Committee of Shanghai Jiaotong University School of Medicine (license number: A‐2019‐041).

## Supporting information

Supporting information.

Supporting information.

Supporting information.

Supporting information.

Supporting information.

## Data Availability

The data that support the findings of this study are available on request from the corresponding author. The data are not publicly available due to privacy or ethical restrictions.
