# Supplementary figures and images for: Loss of SENP3 mediated the formation of nasal polyps in nasal mucosal inflammation by increasing alternative activated macrophage
Source: Immun Inflamm Dis. 2023 Feb 9;11(2):e781. doi: 10.1002/iid3.781 (PMC9910171; doi:10.1002/iid3.781)

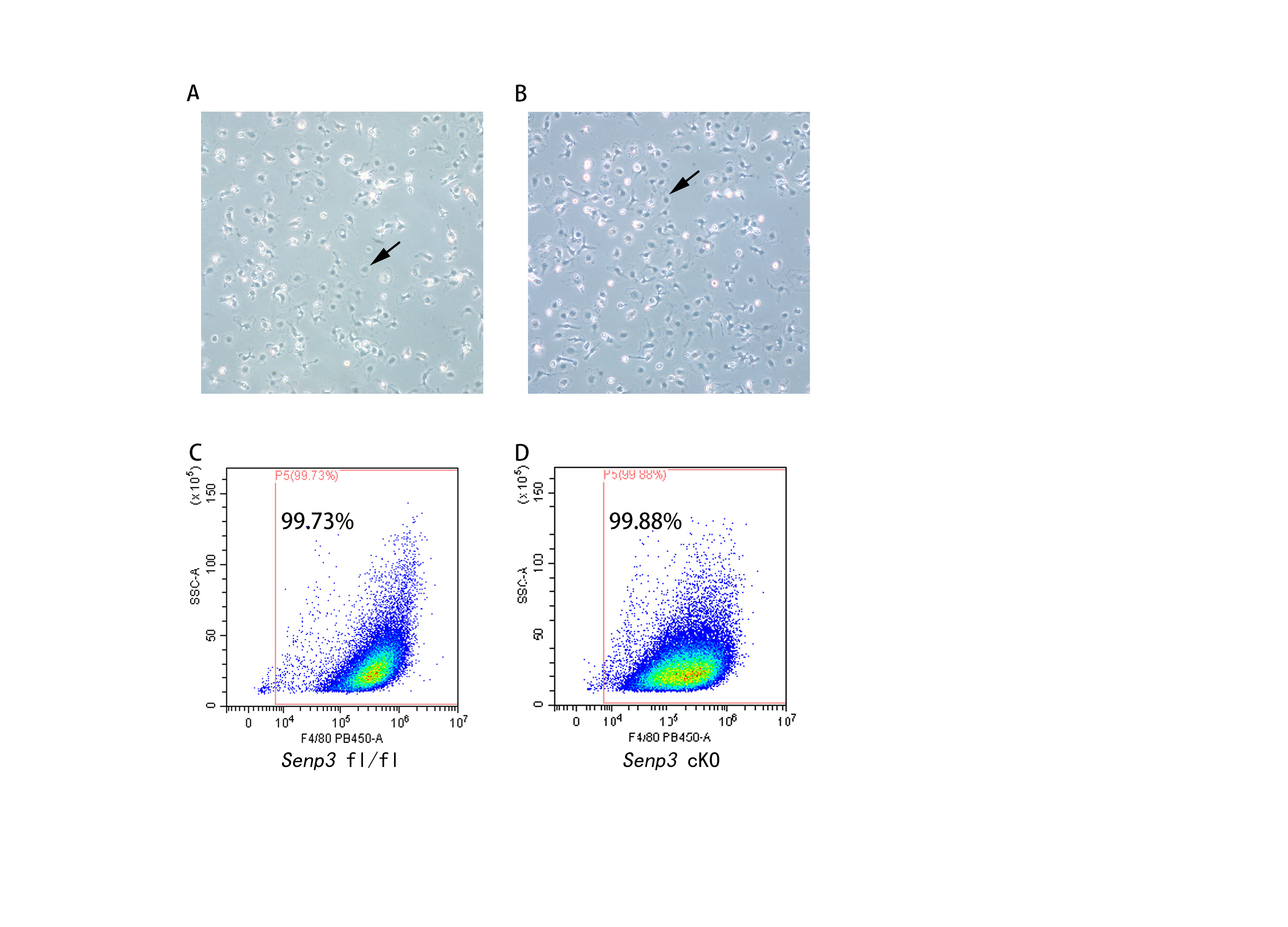

Supplement: Supplementary file 1 — Supporting information. [file IID3-11-e781-s003.jpg]

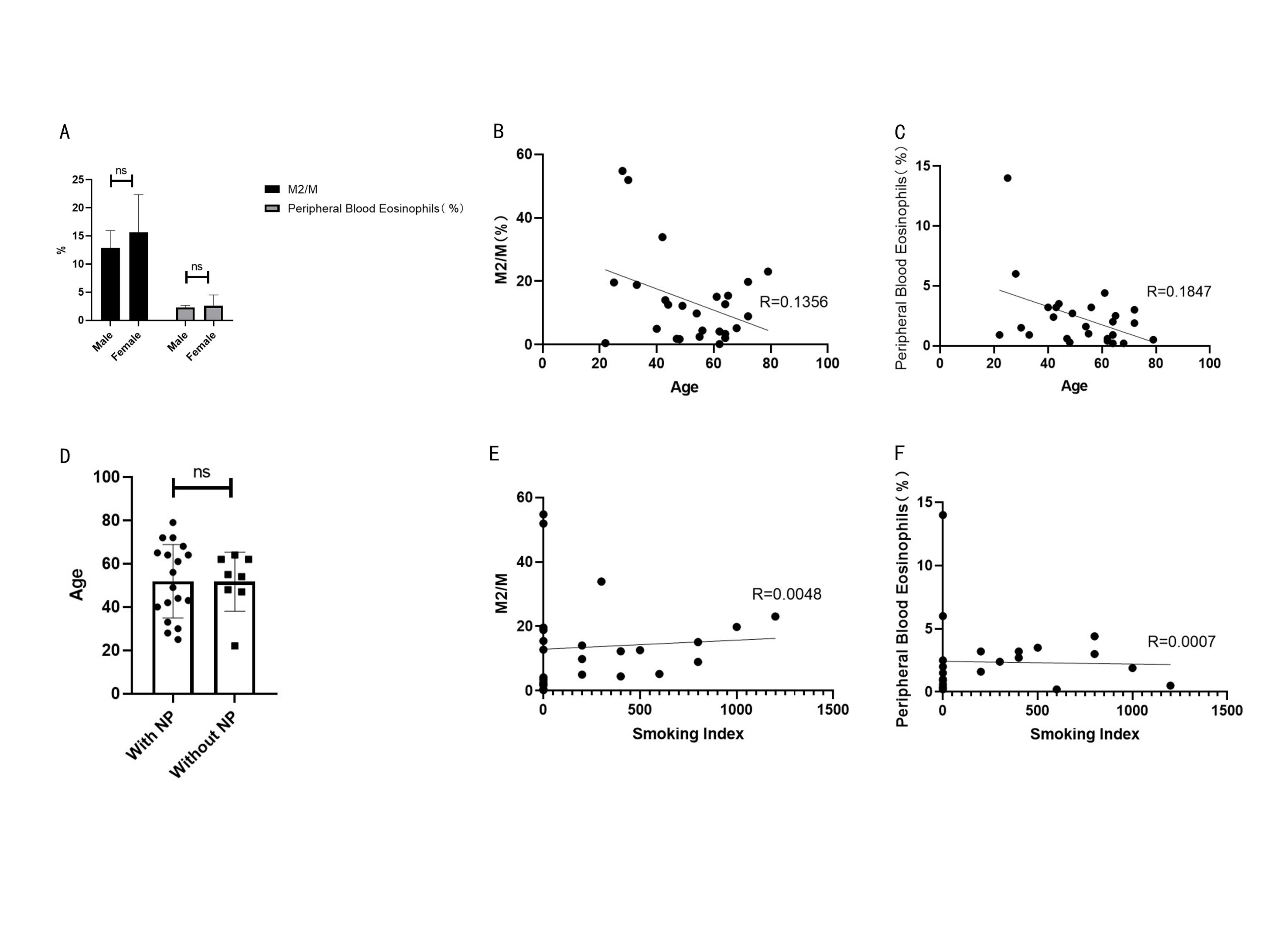

Supplement: Supplementary file 2 — Supporting information. [file IID3-11-e781-s002.jpg]

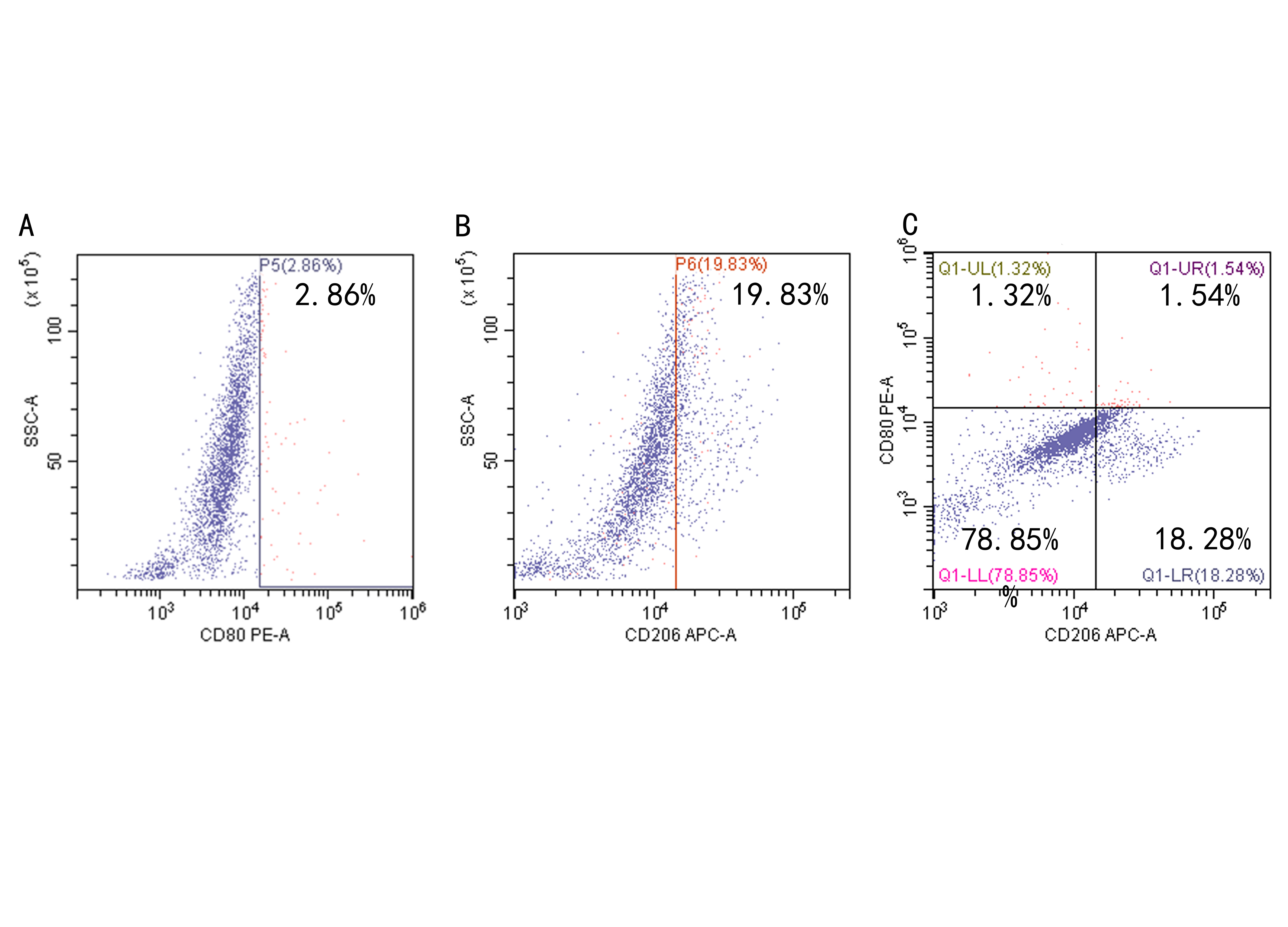

Supplement: Supplementary file 3 — Supporting information. [file IID3-11-e781-s005.jpg]

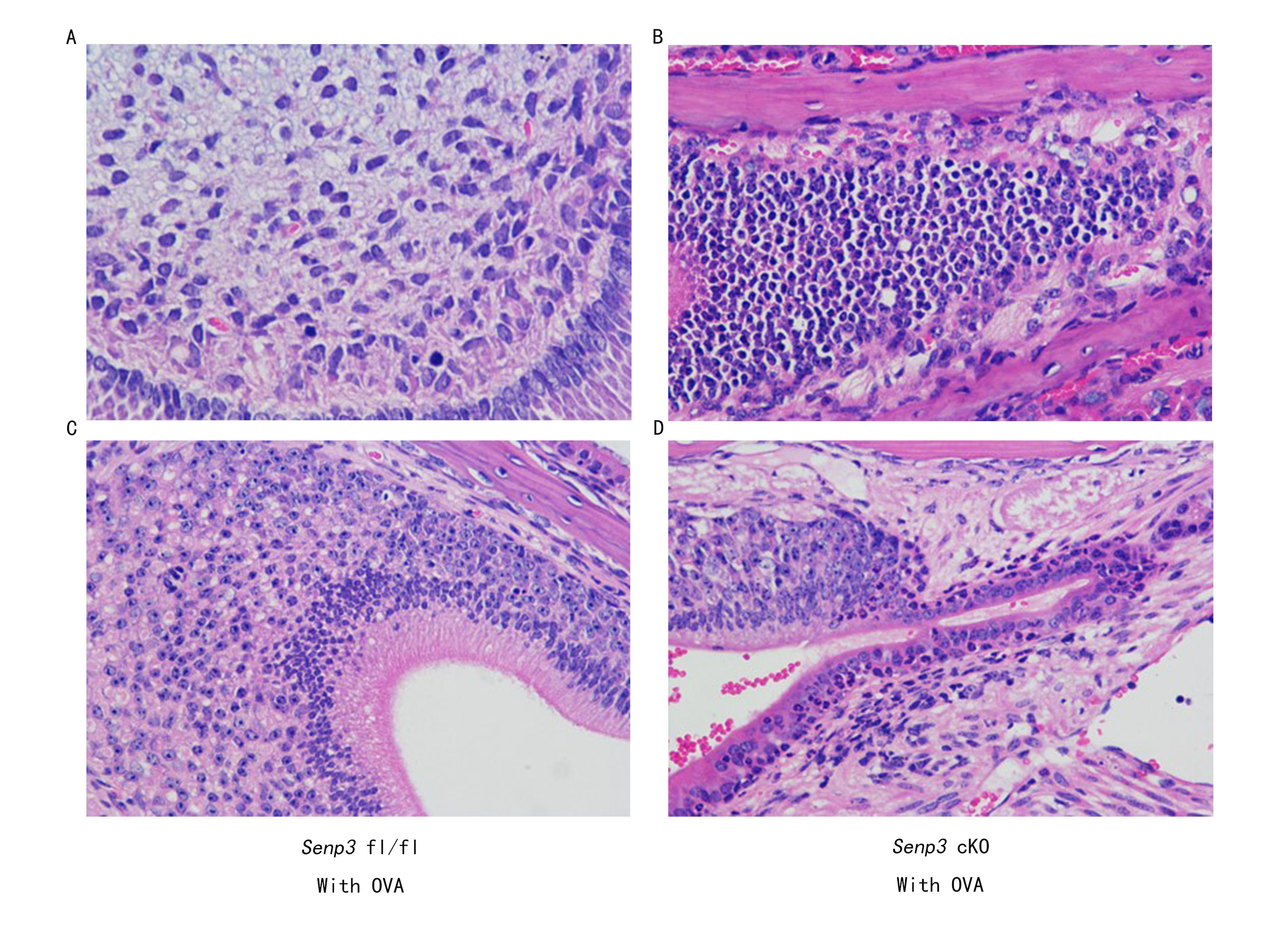

Supplement: Supplementary file 4 — Supporting information. [file IID3-11-e781-s001.jpg]

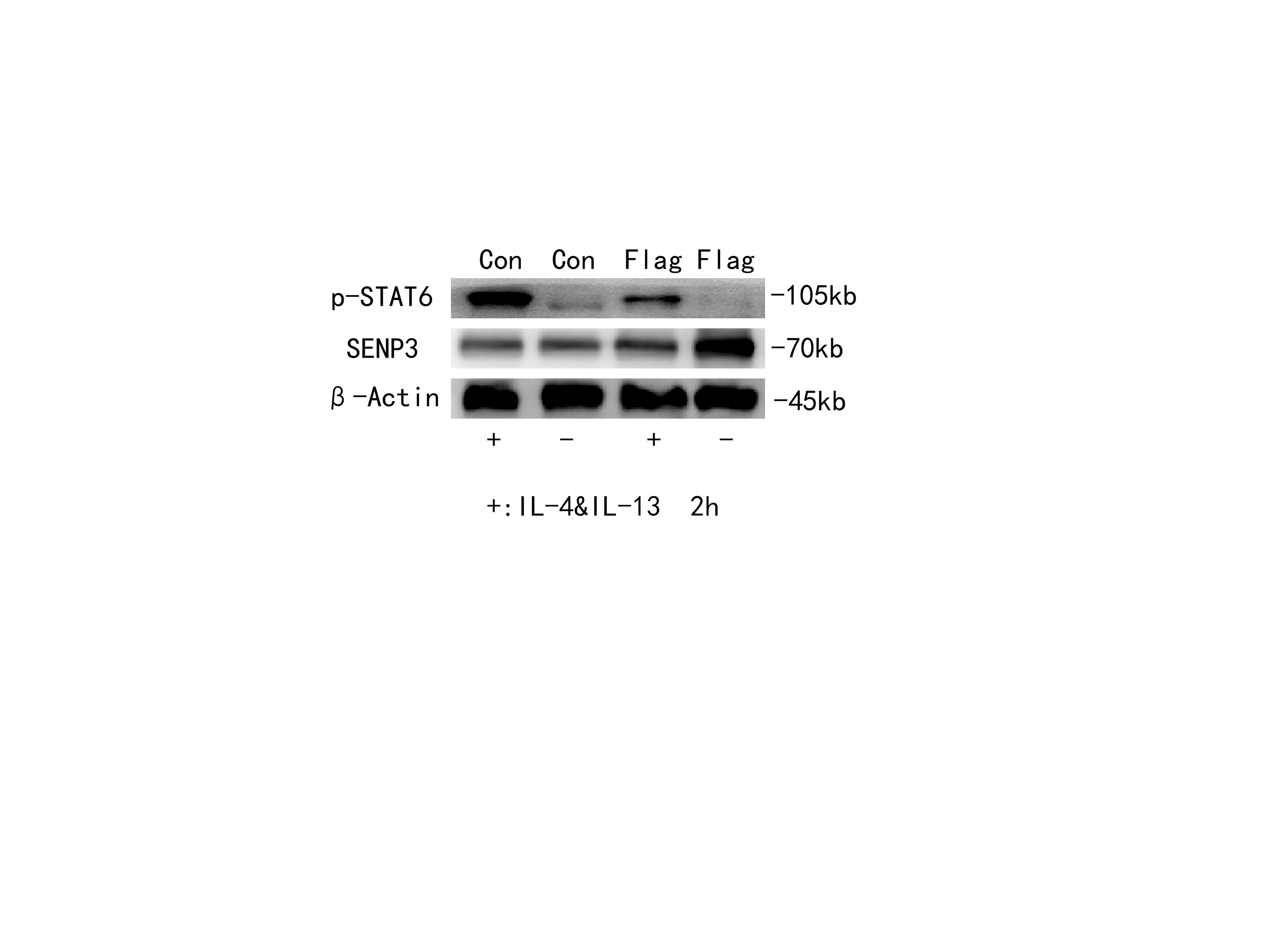

Supplement: Supplementary file 5 — Supporting information. [file IID3-11-e781-s004.jpg]
